# Retinal Vascular Tortuosity Index Change after Idiopathic Epiretinal Membrane Surgery: Does Internal Limiting Membrane Peeling Affect Retinal Vascular Tortuosity?

**DOI:** 10.3390/diagnostics13040797

**Published:** 2023-02-20

**Authors:** Özge Yanık, Pınar Aydın Ellialtıoğlu, Sibel Demirel, Figen Batıoğlu, Emin Özmert

**Affiliations:** Department of Ophthalmology, Ankara University School of Medicine, 06620 Ankara, Turkey

**Keywords:** idiopathic epiretinal membrane, internal limiting membrane, optical coherence tomography angiography, retinal vascular tortuosity index

## Abstract

Background: Idiopathic epiretinal membrane (iERM) surgery is one of the most commonly performed vitreoretinal surgeries, and the issue of internal limiting membrane (ILM) peeling in ERM surgery is still controversial. The aims of this study are to evaluate the changes in retinal vascular tortuosity index (RVTI) after pars plana vitrectomy for the iERM using optical coherence tomography angiography (OCTA) and to assess whether ILM peeling has an additional effect on RVTI reduction. Methods: This study included25 eyes of 25 iERM patients who underwent ERM surgery. The ERM was removed without ILM peeling in 10 eyes (40.0%), and the ILM was peeled in addition to the ERM in 15 eyes (60.0%). The existence of the ILM after ERM peeling was checked with second staining in all eyes. Best corrected visual acuity (BCVA) and 6 × 6 mm en-face OCTA images were recorded before surgery and at the first month postoperatively. A skeleton model of the retinal vascular structure was created following Otsu binarization of en-face OCTA images using ImageJ software (1.52U). RVTI was calculated as the ratio of each vessel length to its Euclidean distance on the skeleton model using the Analyze Skeleton plug-in. Results: The mean RVTI declined from 1.220 ± 0.017 to 1.201 ± 0.020 (*p* = 0.036) in eyes with ILM peeling and from 1.230 ± 0.038 to 1.195 ± 0.024 in eyes without ILM peeling (*p* = 0.037). There was no difference between the groups in terms of postoperative RVTI (*p* = 0.494). A statistically significant correlation was found between postoperative RVTI and postoperative BCVA (rho = 0.408, *p* = 0.043). Conclusions: The RVTI is an indirect indicator of the traction created by the iERM on retinal microvascular structures, and it was effectively reduced after iERM surgery. The postoperative RVTIs were similar in cases who underwent iERM surgery with or without ILM peeling. Therefore, ILM peeling may not have an additive effect on the loosening of microvascular traction and thus may be reserved for recurrent ERM surgeries.

## 1. Introduction

Epiretinal membrane (ERM) is a common vitreoretinal interface disease characterized by abnormal non-vascular fibro-cellular tissue proliferation on the surface of the internal limiting membrane (ILM) [1]. The etiology of the ERM is most commonly idiopathic (iERM) predominantly affecting patients over 50 years of age [2], but ERMs may develop secondary to other ocular disorders such as diabetic retinopathy, retinal vein occlusion, ocular inflammatory disease, trauma, intraocular surgery, intraocular tumors, retinal dystrophies and retinal tear or detachment [3,4,5]. According to the results of the Beaver Dam Eye Study, its prevalence was reported as high as 34.1% using spectral domain optical coherence tomography (SD-OCT) [6].

The initiating event or factors leading to the formation of an iERM is still unclear. ERMs are composed of an extracellular matrix (collagen, laminin, fibronectin, vitronectin, etc.) and cells including glial cells, neurites, retinal pigment epithelium, immune cells, and fibrocytes [7]. A posterior vitreous detachment is observed in approximately 60% to 90% of patients at the time of the ERM diagnosis [7]. Therefore, the proliferation of glial cells entering the retinal surface during a posterior vitreous detachment or partial separation of the posterior hyaloid is the most widely accepted pathogenetic hypothesis [8,9]. In the absence of posterior vitreous detachment, glial cells may grow through the posterior hyaloid, which then in turn becomes incorporated into the membrane [10]. Histopathological studies also showed the myofibroblastic transdifferentiation of retinal glial and pigment epithelial cells on the inner retinal surface [11,12].

The accumulation of myofibroblast-like cells and extracellular matrix proteins is responsible for the contractile properties of ERMs [12]. Hyalocytes were also shown to have a role in the enhancement of ERM contractility [13]. In addition, alterations in the expression of surface proteins contribute to the membrane contractility. It was shown that a reduction in glial fibrillary acidic protein and increase in a-smooth-muscle actin are associated with high contractility [14]. Therefore, ERMs have the potential to affect the microarchitecture of the macula causing loss of the foveal contour, disruption of the inner and outer retinal layers, and increase in vascular tortuosity due to contraction of the membrane [15,16,17]. Although most of the cases are asymptomatic with slowly progressive ERMs without requiring any intervention [18], visual complaints including reduced visual acuity, blurred vision, and metamorphopsia start to occur with the increase in disruption of the retinal layers and the traction on the retina. When the ERM becomes symptomatic causing visual decrease and/or disturbances, vitreoretinal surgery is the only possible treatment option to restore the macular structure, providing removal of the membrane and release of retinal traction. Most of the vitreoretinal surgeons prefer to remove the ILM in addition to the ERM. However, the need for ILM peeling in routine ERM surgery is still under debate. Despite reduced ERM recurrence rates with ILM peeling [19,20], improvements in retinal thickness are limited [21], and functional outcome does not differ [22]. Furthermore, ILM peeling has a potential risk for Müller cell damage [23]. Therefore, it may be a more rational approach to decide additional ILM peeling considering these advantages and disadvantages. Analyses of novel quantitative parameters such as retinal vascular tortuosity index (RVTI) and foveal acircularity index may provide relevant outcomes to evaluate its additive effect on the restoration of macular microarchitecture.

Optical coherence tomography angiography (OCTA) is a new imaging method that depends on the movement of erythrocytes on a stationary background providing high-quality images of retinal and choroidal vasculature [24]. This technology allows the evaluation of superficial/deep capillary plexi and foveal avascular zone (FAZ). Its non-invasive nature and capacity to produce highly reliable and reproducible measurements make OCTA an important monitoring tool in the evaluation of retinal microvascular changes [25]. A recent study has successfully used it to evaluate retinal vascular distortion in eyes with the ERM and to observe changes in retinal vascular distortion after surgery [16].

The aims of this study are to assess the changes in RVTI after pars plana vitrectomy (PPV) surgery for the iERM using OCTA and to evaluate whether internal limiting membrane (ILM) peeling has an additional effect on RVTI. To the best of our knowledge, there is no study showing whether ILM peeling has an effect on the reduction in traction on microvascular structures in cases with the iERM.

## 2. Materials and Methods

This was a single-center study including 25 eyes of 25 consecutive patients diagnosed with an iERM who underwent PPV for epiretinal membrane peeling between 2020 and 2022 at the Department of Ophthalmology, Ankara University School of Medicine. 

### 2.1. Patient Selection

Inclusion criteria were having PPV surgery for a symptomatic iERM and availability of preoperative and postoperative 1-month SD-OCT and OCTA images. The preoperative multimodal images of each study eye, including multicolor fundus photography, infrared reflectance, and SD-OCT images were also evaluated to confirm the tractional iERM.

The exclusion criteria were the presence of any secondary cause of ERM, pseudohole, tractional or degenerative lamellar macular holes, lamellar hole-associated epiretinal proliferation, additional ocular disease (uveitis, glaucoma, angioid streaks, etc.), any systemic disease that has the potential to affect the retina and choroid (diabetes mellitus, sickle cell anemia, etc.), media opacity preventing adequate imaging, high refractive error ≥5.00 D spherical equivalent, low-quality OCTA images below 6/10 scanning quality, and a history of retinal laser photocoagulation and any previous intraocular surgery with the exclusion of uncomplicated phacoemulsification.

Demographic and clinical data were collected from the medical records at preoperative and postoperative 1-month visits. All patients underwent a full ophthalmic examination including best corrected visual acuity (BCVA), intraocular pressure measurement with a handheld tonometer (Tono-Pen AVIA^®^, Reichert Technologies, New York, NY, USA), slit lamp biomicroscopy, a dilated fundus examination, and axial length measurement with noncontact partial coherence laser interferometry (IOLMaster^®^ 500; Carl Zeiss Meditec, Jena, Germany). BCVA was measured using the Early Treatment in Diabetic Retinopathy Study (ETDRS) charts and converted to a logarithm of the minimum angle of resolution (LogMAR) unit. Ophthalmological examination findings and OCTA images of the cases at the preoperative and postoperative 1st month were analyzed.

### 2.2. Surgical Procedure

Transconjunctival sutureless three-port PPV was performed using a 25G, 7500 cuts-per-minute device (Constellation Vision System, Alcon Surgical, Irvine, CA, USA) by two experienced vitreoretinal surgeons (SD, FB). A wide-angle viewing system (EIBOS 2, Haag-Streit Surgical) and high magnification contact lens (HR Direct High Mag Surgical Lens, Volk Optical, Inc., Mentor, OH, USA) were used for surgical visualization. Trocars were inserted 3.5 mm from the limbus. The vitreous humor was stained with intravitreal triamcinolone, and the posterior hyaloid was separated. The ERM/ILM was stained with intravitreal MembraneBlue-Dual (Combination of 0.025% brilliant blue and 0.15% trypan blue, DORC International, Zuidland, The Netherlands) and was peeled circumferentially using forceps. The ERM was removed without ILM peeling in 10 eyes (40.0%) and the ILM was peeled in addition to the ERM in 15 eyes (60.0%). The existence of the ILM after ERM peeling was checked with second dye staining in all eyes.

### 2.3. Multimodal Imaging and Quantitative Measurements

SD-OCT (Spectralis, Heidelberg Engineering Inc., Germany) and OCTA (Avanti RT Vue XR^®^ with AngioVue^®^ software; Optovue Inc., Fremont, CA, USA) were performed in all patients after pupil dilation. The severity of the ERM was graded according to the ERM OCT staging scheme defined by Govetto et al. [17]. Presence of the cotton ball sign, a round or diffuse hyper-reflective area between the ellipsoid zone and the cone outer segment tip line at the central fovea was also evaluated [26]. Preoperative and postoperative central subfield thicknesses (CSTs), the mean thickness of the macula in the central 1 mm ETDRS grid, were measured by the mapping software of SD-OCT.

The evaluated OCTA parameters included FAZ area, FAZ perimeter, foveal vessel density within a 300 μm width around the FAZ, and acircularity index. Acircularity index was defined as the ratio of the perimeter of the FAZ to the perimeter of a circle with equal area. A perfectly circular shape had an acircularity index equal to 1, with deviations from acircularity leading to an increase in this index. FAZ measurements could not be evaluated in 3 eyes due to due to very dense traction on the fovea.

### 2.4. The Retinal Vascular Tortuosity Index Measurement

**Figure 1:**
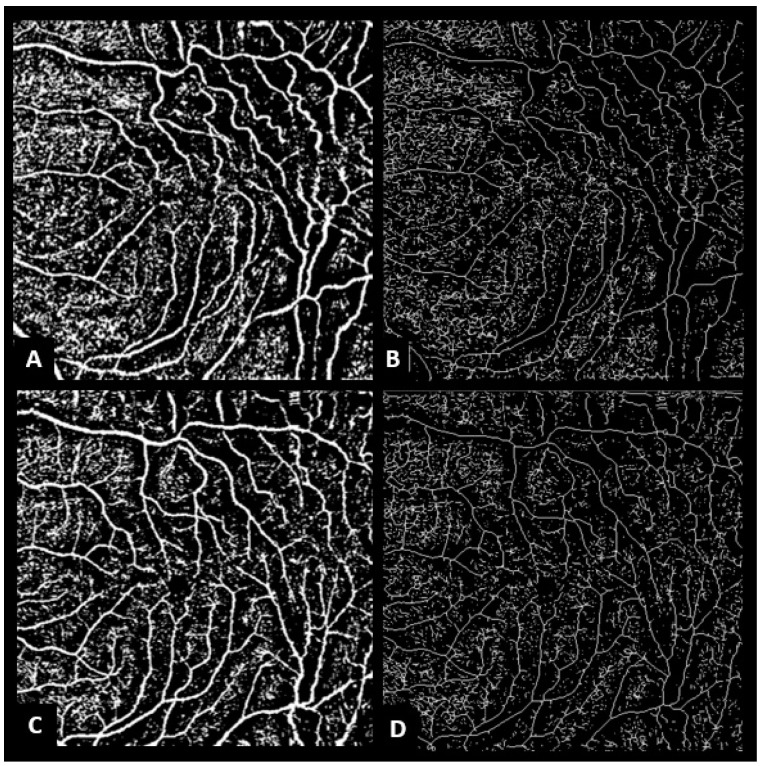
Binarized en-face OCTA images of a 65-year-old female before and after idiopathic epiretinal membrane (iERM) surgery. (**A**) Otsu binarization of en-face OCTA image before iERM surgery. (**B**) Skeleton model of the superficial retinal vascular plexus, retinal vascular tortuosity index was 1.22. (**C**) Otsu binarization of en-face OCTA image after iERM surgery. (**D**) Skeleton model of the superficial retinal vascular plexus, the retinal tortuosity index regressed to 1.17 after surgery.

The RVTI measurements were performed using ImageJ program version 1.52 u 64-bit Java 1.80_112 (Wayne Rasband, National Institutes of Health, Bethesda, MD, USA, https://imagej.nih.gov/ij accessed on 4 April 2020). In brief, 6 × 6 mm en-face OCTA images were converted into 8-bit grayscale images. Then, the Otsu method was used to binarize the OCTA image. A skeleton model of the superficial retinal vascular plexus was created (Figure 1). RVTI was calculated as the ratio of each vessel length to its Euclidean distance on the skeleton model using the Analyze Skeleton plug-in [27].

The primary outcome measures of the present study were the changes in the RVTI and FAZ parameters. The secondary outcome measure of this study was the correlation of postoperative BCVA with postoperative RVTI and FAZ parameters.

### 2.5. Statistical Analysis

Statistical Package for the Social Sciences software (version 15.0) was used for the analyses. The variables were investigated using visual (histograms, probability plots) and analytic methods (Shapiro–Wilk test) to test the distribution of the data. Continuous variables were expressed as the mean ± standard deviation (SD). Categorical variables were expressed as the number of observations and/or percentages. Depending on the distribution of the data, the baseline and postoperative 1st month data were compared using the paired sample *t* test or the Wilcoxon signed rank test. Differences between the ILM-peeled and unpeeled groups were compared using the independent sample *t* test or the Mann–Whitney U test. The relationship between BCVA and RVTI was evaluated using the Spearman correlation test. Results for *p* < 0.05 were considered statistically significant.

## 3. Results

This study included 25 eyes of 25 consecutive patients with a mean age of 64.8 ± 4.0 years. Fifteen of the patients (60%) were female and 10 (40%) were male. According to the ERM-OCT staging scheme, three eyes (12.0%) had stage 2, 14 eyes (56.0%) had stage 3, and eight eyes (32.0%) had stage 4 disease. The cotton ball sign was observed in three eyes (12.0%). The mean BCVA increased from 0.48 ± 0.22 LogMAR to 0.18 ± 0.15 LogMAR (*p* < 0.001), the mean RVTI decreased from 1.224 ± 0.027 to 1.199 ± 0.021 (*p* = 0.004), and the mean CST decreased from 477.3 ± 95.9 µm to 423.7 ± 59.8 µm (*p* = 0.001) in the postoperative first month (Table 1). 

**Figure 2:**
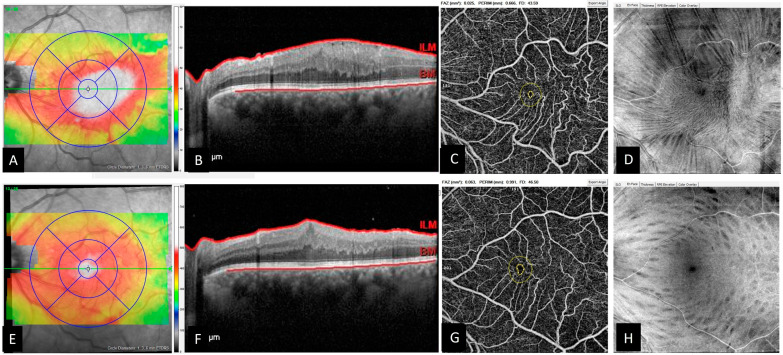
OCT and en-face OCTA images of a 67-year-old male before and after idiopathic epiretinal membrane (iERM) surgery. (**A**) Preoperative retinal thickness map calculated by the software of SD-OCT device showed the increased retinal thickness. The central subfield thickness was 545 µm. (**B**) The horizontal B-scan SD-OCT passing through the fovea revealed a stage 3 epiretinal membrane: The reflectivity of the ectopic inner foveal layers is similar to that of the inner nuclear layer. (**C**) The en-face OCTA image shows measured foveal parameters including foveal avascular zone (FAZ) area (0.025 mm^2^), FAZ perimeter (0.666 mm), and foveal vessel density (43.59%). Note the increased retinal vessel tortuosity. (**D**) En-face OCT reveals the surface traction. (**E**) Postoperative retinal thickness map showed the reduction in retinal thickness. The central subfield thickness was 511 µm. (**F**) The horizontal B-scan OCT passing through the fovea revealed an absence of foveal pit. (**G**) After iERM surgery, all the measured foveal parameters increased (FAZ area = 0.063 mm^2^, FAZ perimeter = 0.991 mm, and foveal vessel density (46.50%). (**H**) The surface traction was relieved together with decreased vessel tortuosity. Note the inner retinal dimples coursing along the path of the nerve fiber layer caused by the internal limiting membrane peeling.

Regarding measured FAZ parameters, there were no significant differences between preoperative and postoperative mean FAZ area (0.089 ± 0.07 mm^2^ vs. 0.085 ± 0.04 mm^2^, *p* = 0.516), mean FAZ perimeter (1.201 ± 0.36 mm vs. 1.161 ± 0.28 mm, *p* = 0.548), and mean foveal vessel density within a 300 μm width around the FAZ (44.2 ± 8.29 vs. 46.5 ± 5.86%, *p* = 0.277) (Figure 2). The mean FAZ acircularity index significantly decreased from 1.22 ± 0.11 to 1.16 ± 0.10 (*p* = 0.022) after PPV surgery. When FAZ measurements were compared between the two ILM groups, no difference was found between preoperative and postoperative mean FAZ measurements. 

Comparing ILM groups, the mean axial length in eyes with ILM peeling was 23.64 ± 0.96 mm (21.86–25.16 mm), and the mean axial length in in eyes without ILM peeling was 23.59 ± 0.78 mm (21.97–24.54 mm) (*p* = 0.728). The mean RVTI declined from 1.220 ± 0.017 to 1.201 ± 0.020 (*p* = 0.036) in eyes with ILM peeling and from 1.230 ± 0.038 to 1.195 ± 0.024 in eyes without ILM peeling (*p* = 0.037). There was no difference between the groups in terms of postoperative mean RVTI (*p* = 0.494) (Table 2).

**Figure 3:**
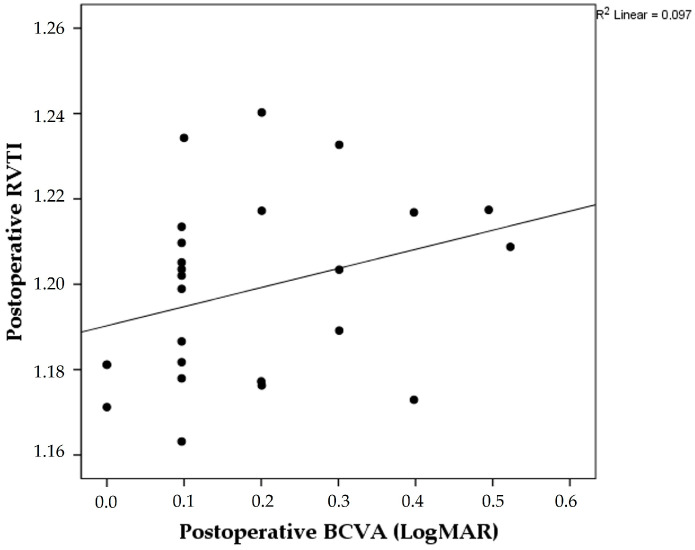
Scatter plot graph showing the correlation of postoperative best-corrected visual acuity (BCVA) scores and postoperative retinal vascular tortuosity index (RVTI).

Considering the relationship of evaluated parameters with postoperative BCVA scores, the only statistically significant correlation was found between postoperative RVTI and postoperative BCVA (rho = 0.408, *p* = 0.043) (Figure 3).

## 4. Discussion

The RVTI is an indirect indicator of the traction created by the ERM on retinal microvascular structures. This study showed that the RVTI was significantly reduced after ERM peeling, whether the ILM was peeled or not. No significant difference was observed between the ILM peeled and unpeeled groups in terms of postoperative RVTI. In addition, the statistically significant correlation between postoperative RVTI and BCVA suggested that traction on retinal microvascular structure had a negative effect on BCVA.

Normal retinal blood vessels are straight or gently curved [28]. Retinal vessel tortuosity was described as the presence of retinal vascular curliness and is usually evaluated clinically at the time of fundus examination [27]. Many retinal pathologies, systemic diseases (diabetic retinopathy, cerebrovascular disease, stroke, and ischemic heart disease, etc.) and genetic disorders (familial retinal arteriolar tortuosity, Fabry disease, etc.) may be associated with retinal vessel tortuosity [29,30,31]. The objective quantification of vessels tortuosity in retinal images may have a diagnostic value for these disorders and it may also be an important monitoring tool. Therefore, many studies have focused on the quantitative measurement of retinal vascularity in these disorders [32]. Although fundus photographs or fluorescein angiography images were used initially for the evaluation of vessels tortuosity [28,33], recent publications have preferred OCTA because of its superior ability to demonstrate retinal microvasculature [16,27,32]. Lee et al. defined an automated measurement method using en-face OCTA slabs and ImageJ [32].

Centripetal tractional force is believed to be the main pathogenesis of the ERM [34,35]. The tractional effect of the ERM on the retinal surface alters the macular microstructure including surface wrinkling, winding corkscrew vessels, or major vessel straightening and crowding and subsequently thickens the macula, leading to the development of ectopic inner foveal layers and disruption of the outer and inner retinal layer causing a decrease in BCVA [17,36,37]. Different methods have been described in the literature to evaluate the severity of this traction. Especially, retinal vessels have become the focus of interest in the quantitative evaluation of the tangential contraction [16,35,36,38,39,40,41]. Many studies have preferred to measure the moving distance of retinal vessels caused by the ERM [35,36,38,39,40,41,42]. For this purpose, fundus photographs [36,39,40], infrared reflectance images [35,41], and fundus autofluorescence (FAF) imaging [38,42] were used to measure the movements of the retinal vessels manually. Kofod et al. showed that retinal vessel movement was associated with BCVA worsening in eyes with an ERM [35]. Retinal vessel printings, hyperautofluorescent lines indicating the original location of the retinal vessels on fundus autofluorescence imaging, were reported to provide important data about the severity and direction of the tangential traction [42]. Rodrigues et al. analyzed fundus autofluorescence imaging for evidence of retinal displacement and reported that the vertical distance between the posterior veins increased after ERM surgery [38]. The most recent one of those studies was conducted by Ichikawa et al. [41]. They evaluated the relationship of metamorphopsia and tangential retinal displacement after epiretinal membrane surgery. To evaluate the tangential retinal displacement, they used manual horizontal and vertical drawings passing through the fovea on infrared reflectance imaging. The distance between each set of intersections was manually measured with the caliper function embedded. Although that study provided significant data about the relationship of the degree of metamorphopsia and the tangential retinal displacement, the manual measurement of the retinal displacement was an important limitation. 

OCTA was shown to be an appropriate tool to evaluate retinal vascular system distortions in eyes with an ERM [16,43,44]. Mastropasqua et al. conducted the first study evaluating not only the vessel tortuosity but also perfusion density, vessel length density, and vessel diameter index of superficial capillary plexus in both the parafoveal and perifoveal areas [43]. They used Lee et al.’s method [32] for the calculation of vessel tortuosity and observed a significant reduction in the retinal vessel tortuosity in the parafoveal area at the postoperative period [43]. However, since ILM peeling was also performed in all cases in that study, it was not possible to make a conclusion about ILM peeling. The second study in the current literature was conducted by Miyazawa et al. [16]. In that study, the authors manually picked a retinal vessel in each of the four quadrants of the vessels descending to the macula. They divided the actual vessel length in the vessel section by the direct vessel branching point distance in the four quadrants and reported that the ratio of vessel length to the shortest distance between two branches decreased significantly after ERM surgery. Although that study was able to quantitatively measure vascular tortuosity in an ERM, it had some limitations such as use of the manual method and not being able to evaluate all macular vessels. To the best of our knowledge, there is no study in the current literature reporting the impact of ILM peeling on microvascular structures in addition to ERM removal and answering whether we can obtain any benefit regarding anatomy and functional outcome if we peel both in the same surgery. Our study is the first using the automated method described by Lee et al. [32] for the evaluation of the additive effect of ILM peeling on RVTI in iERM cases. This method enables quantitative evaluation of the entire macular vessels. Although ILM peeling is generally considered a fundamental step in macular hole repair, its role in ERM surgery remains controversial. Many authors have argued that the ILM should be removed to reduce the likelihood of ERM recurrence [19,20]. Despite this, peeling of the ILM is associated with inner retinal dimpling and greater microscotomas [45,46]. In terms of visual outcomes, it is suggested that there is no additional benefit when comparing eyes with ILM peeling versus eyes without ILM peeling [22]. ILM peeling may reduce the incidence of recurrent ERMs, but many recurrent ERMs may not be visually significant [47]. Therefore, some authors have suggested double peeling only for recurrent ERMs [48]. Our study presents quantitative evidence about the changes in retinal microvascular structures and provides important data that peeling the only ERM may be sufficient to relieve microvascular traction. According to the results of our study, there was no difference neither in BCVA outcomes nor anatomical vascular improvement between groups.

The FAZ is typically circular or elliptical in healthy individuals, with deviations from this shape including a small FAZ area and abnormal circularity/roundness of the FAZ often seen in ERMs [49,50]. Because of the horizontal direction of retinal nerve fibers, ERM contracts more vertically, so the shape of the FAZ becomes horizontally long in most of the ERM eyes [51]. Prior studies have qualitatively demonstrated changes in FAZ parameters after ERM surgery. Most of the authors showed FAZ enlarged and became more circular postoperatively [44,52,53,54,55], but in some, it did not change [50] or decreased [16]. Regarding FAZ acircularity, it was reported that uneven forces from epiretinal membrane led to a higher acircularity index compared to a lower acircularity index caused by the homogenous forces from internal limited membrane [56]. In our study, although there was no change in the mean FAZ area, mean FAZ parameter, and mean foveal vessel density within a 300 μm width around the FAZ, the decrease in the acircularity index after iERM surgery indicated that the FAZ became more circular, as in healthy cases, with the elimination of the tractional effect of ERMs. A significant improvement in the FAZ acircularity index was observed in the group in which the only the ERM was peeled off. Furthermore, the ILM peeling process may cause a foveal displacement of capillaries due to the increased retinal elasticity [57]. Kumagai et al. reported a centripetal movement of the inner retinal layer with a centripetal shift of foveal capillaries after ILM peeling [58]. Therefore, the change in the foveal acircularity index in the group in which the ILM was also peeled may not have reached the limit of statistical significance.

This study was conducted as a pilot study. The strength of this study is the measurement of the RVTI in an automated manner using en-face OCTA images. The major limitations of this study are its retrospective nature, small number of iERM cases, and the short postoperative follow-up. Therefore, these findings are needed to be supported by larger sample groups. In addition, studies with longer follow-up periods are needed to further evaluate the changes in these findings over time.

## 5. Conclusions

RVTI has effectively reduced after iERM surgery. The significant correlation between postoperative RVTI and postoperative BCVA suggested that decreased traction on the retinal surface may be associated with better functional outcome. The postoperative RVTIs were similar in cases that underwent iERM surgery with or without ILM peeling. Therefore, ILM peeling reduces the risk of recurrences after first surgery, but it may not have an additive effect on the loosening of microvascular traction or functional outcome over ERM peeling only.

## Figures and Tables

**Table 1:** Comparison of the mean best corrected visual acuities, retinal vascular tortuosity indices, central subfield thicknesses, and FAZ parameters in patients undergoing surgery for idiopathic epiretinal membrane before and after 25G pars plana vitrectomy.

	Preoperative Mean ± SD (Min–Max)	Postoperative Mean ± SD (Min–Max)	*p* Value
**Patients, *n* = 25**			
BCVA (LogMAR)	0.48 ± 0.22 (0.20–1.00)	0.18 ± 0.15 (0.00–0.52)	<0.001 ^a^
RVTI	1.224 ± 0.027 (1.19–1.31)	1.199 ± 0.021 (1.16–1.24)	0.004 ^a^
Central subfield thickness, µm	477.3 ± 95.9 300–691	423.7 ± 59.8 306–515	0.001
FAZ area, mm^2^	0.089 ± 0.07 0.027–0.249	0.085 ± 0.04 0.034–0.201	0.516
FAZ perimeter, mm	1.201 ± 0.36 0.698–1.964	1.161 ± 0.28 0.724–1.800	0.548
Foveal vessel density, %	44.2 ± 8.29 27.6–58.3	46.5 ± 5.86 37.1–57.6	0.277
FAZ acircularity index	1.22 ± 0.11 1.06–1.38	1.16± 0.10 1.06–1.45	0.022
**ERM, *n* = 10**			
BCVA (LogMAR)	0.56 ± 0.26 (0.20–1.00)	0.25 ± 0.16 (0.00–0.49)	0.003 ^b^
RVTI	1.230 ± 0.038 (1.19–1.31)	1.195 ± 0.024 (1.16–1.24)	0.037 ^a^
Central subfield thickness, µm	473.5 ± 106.0 (338–691)	425.7 ± 72.1 (297–511)	0.017
FAZ area, mm^2^	0.081 ± 0.06 (0.040–0.249)	0.090 ± 0.03 0.063–0.144	0.109
FAZ perimeter, mm	1.187 ± 0.33 (0.918–1.964)	1.197 ± 0.21 0.961–1.542)	0.594
Foveal vessel density, %	41.6 ± 9.69 (27.6–57.4)	44.7 ± 6.28 (37.1–53.7)	0.374
FAZ acircularity index	1.25 ± 0.08 (1.11–1.36)	1.14 ± 0.06 (1.08–1.26)	0.028
**ERM + ILM, *n* = 15**			
BCVA (LogMAR)	0.42 ± 0.17 (0.20–0.70)	0.14 ± 0.13 (0.00–0.52)	0.005 ^a^
RVTI	1.220 ± 0.017 (1.20–1.26)	1.201 ± 0.020 (1.17–1.23)	0.036 ^a^
Central subfield thickness, µm	479.8 ± 92.4 (300–588)	422.4 ± 52.8 (338–515)	0.023
FAZ area, mm^2^	0.095 ± 0.07 0.027–0.225	0.082 ± 0.05 0.034–0.201	0.675
FAZ perimeter, mm	1.211 ± 0.39 (0.698–1.925)	1.136 ± 0.32 0.724–1.800	0.221
Foveal vessel density, %	46.0 ± 6.99 (35.5–58.3)	47.7 ± 5.47 39.0–57.6	0.650
FAZ acircularity index	1.21 ± 0.12 (1.06–1.38)	1.17 ± 0.13 1.06–1.45	0.311

BCVA: best corrected visual acuity, ERM: epiretinal membrane, ILM: internal limiting membrane; RVTI: retinal vascular tortuosity index. ^a^ Wilcoxon signed-rank test, ^b^ paired samples *t* test.

**Table 2:** Comparison of the preoperative and postoperative mean best corrected visual acuities, retinal vascular tortuosity indices, central subfield thicknesses, and FAZ parameters in patients according to the additional internal limiting membrane peeling.

	ERM, *n* = 10 Mean ± SD (Min–Max)	ERM + ILM, *n* = 15 Mean ± SD (Min–Max)	*p* Value
**Preoperative**			
BCVA (LogMAR)	0.56 ± 0.26 (0.20–1.00)	0.42 ± 0.17 (0.20–0.70)	0.133 ^a^
RVTI	1.230 ± 0.038 (1.19–1.31)	1.220 ± 0.017 (1.20–1.26)	0.956 ^a^
Central subfield thickness, µm	473.5 ± 106.0 (338–691)	479.8 ± 92.4 (300–588)	0.542 ^a^
FAZ area, mm^2^	0.081 ± 0.06 (0.040–0.249)	0.095 ± 0.07 0.027–0.225	0.973 ^a^
FAZ perimeter, mm	1.187 ± 0.33 (0.918–1.964)	1.211 ± 0.39 (0.698–1.925)	0.973 ^a^
Foveal vessel density, %	41.6 ± 9.69 (27.6–57.4)	46.0 ± 6.99 (35.5–58.3)	0.271 ^a^
FAZ acircularity index	1.25 ± 0.08 (1.11–1.36)	1.21 ± 0.12 (1.06–1.38)	0.616 ^a^
**Postoperative**			
BCVA (LogMAR)	0.25 ± 0.16 (0.00–0.49)	0.14 ± 0.13 (0.00–0.52)	0.085 ^a^
RVTI	1.195 ± 0.024 (1.16–1.24)	1.201 ± 0.020 (1.17–1.23)	0.494 ^b^
Central subfield thickness, µm	425.7 ± 72.1 (297–511)	422.4 ± 52.8 (338–515)	0.868 ^a^
FAZ area, mm^2^	0.090 ± 0.03 0.063–0.144	0.082 ± 0.05 0.034–0.201	0.404 ^a^
FAZ perimeter, mm	1.197 ± 0.21 0.961–1.542)	1.136 ± 0.32 0.724–1.800	0.616 ^a^
Foveal vessel density, %	44.7 ± 6.28 (37.1–53.7)	47.7 ± 5.47 39.0–57.6	0.217 ^a^
FAZ acircularity index	1.14 ± 0.06 (1.08–1.26)	1.17 ± 0.13 1.06–1.45	0.920 ^a^

BCVA: best corrected visual acuity, ERM: epiretinal membrane, ILM: internal limiting membrane; RVTI: retinal vascular tortuosity index. ^a^ Mann–Whitney U test, ^b^ Independent sample *t* test.

## Data Availability

The datasets generated during and/or analyzed during the current study are available from the corresponding author on reasonable request.
